# Mechanisms of Cell Killing Response from Low Linear Energy Transfer (LET) Radiation Originating from ^177^Lu Radioimmunotherapy Targeting Disseminated Intraperitoneal Tumor Xenografts

**DOI:** 10.3390/ijms17050736

**Published:** 2016-05-16

**Authors:** Kwon Joong Yong, Diane E. Milenic, Kwamena E. Baidoo, Martin W. Brechbiel

**Affiliations:** Radioimmune & Inorganic Chemistry Section, Radiation Oncology Branch, National Cancer Institute, National Institutes of Health, 10 Center Drive MSC-1002, Bethesda, MD 20892, USA; halroya@gmail.com (K.J.Y.); milenic.dm71q@nih.gov (D.E.M.); kwamena.baidoo@nih.gov (K.E.B.)

**Keywords:** ^177^Lu-Radioimmunotherapy (RIT), DNA double strand breaks, DNA repair, apoptosis, micrometastatic disease

## Abstract

Radiolabeled antibodies (mAbs) provide efficient tools for cancer therapy. The combination of low energy β^−^-emissions (500 keV_max_; 130 keV_ave_) along with a γ-emission for imaging makes ^177^Lu (T_1/2_ = 6.7 day) a suitable radionuclide for radioimmunotherapy (RIT) of tumor burdens possibly too large to treat with α-particle radiation. RIT with ^177^Lu-trastuzumab has proven to be effective for treatment of disseminated HER2 positive peritoneal disease in a pre-clinical model. To elucidate mechanisms originating from this RIT therapy at the molecular level, tumor bearing mice (LS-174T intraperitoneal xenografts) were treated with ^177^Lu-trastuzumab comparatively to animals treated with a non-specific control, ^177^Lu-HuIgG, and then to prior published results obtained using ^212^Pb-trastuzumab, an α-particle RIT agent. ^177^Lu-trastuzumab induced cell death via DNA double strand breaks (DSB), caspase-3 apoptosis, and interfered with DNA-PK expression, which is associated with the repair of DNA non-homologous end joining damage. This contrasts to prior results, wherein ^212^Pb-trastuzumab was found to down-regulate RAD51, which is involved with homologous recombination DNA damage repair. ^177^Lu-trastuzumab therapy was associated with significant chromosomal disruption and up-regulation of genes in the apoptotic process. These results suggest an inhibition of the repair mechanism specific to the type of radiation damage being inflicted by either high or low linear energy transfer radiation. Understanding the mechanisms of action of β^−^- and α-particle RIT comparatively through an *in vivo* tumor environment offers real information suitable to enhance combination therapy regimens involving α- and β^−^-particle RIT for the management of intraperitoneal disease.

## 1. Introduction

Various approaches using monoclonal antibodies (mAbs) or mAbs conjugated with cytotoxic agents (*i.e.*, radionuclides, drugs, or toxins) have emerged as promising treatment strategies [1] for cancer. Radioimmunotherapy (RIT), the selective targeting of tumor cells with radiolabeled mAbs as vectors to deliver radiation, through delivery of a β^−^- or α-emitting radionuclide, to cancer cells continues to be a burgeoning area of research [2,3]. Trastuzumab, a humanized mAb, has been proven to be a suitable vector for executing RIT targeting HER2 positive tumors [4,5]. Radiolabeled trastuzumab may also enable successful treatment of patients with lower HER2 expression [6,7].

Lutetium-177 (^177^Lu) is a low-energy β^−^-emitter (0.497 MeV_max_) with a relatively short tissue penetration range (max. 1.6 mm). RIT with ^177^Lu possesses advantages *versus* other conventional metallic β^−^-emitting radionuclides (low linear energy transfer (LET)) such as ^90^Y for cancer therapy. These physical properties suggest ^177^Lu to be an appropriate choice for treating small tumor lesions and micrometastases while also limiting normal tissue damage [8,9]. Recent studies have determined an optimal therapy dose and shown the exquisite potential effectiveness of ^177^Lu labeled trastuzumab for treatment of disseminated, HER2 positive, peritoneal disease [10]. Surprisingly, the mechanisms of β^−^-emitter-induced cell killing in disseminated intraperitoneal (i.p.) disease are poorly understood at the molecular level in actual, *in vivo*, tumor tissue.

In contrast to low-LET RIT, e.g., ^177^Lu, α-particle RIT offers far more specific tumor cell killing with less surrounding normal tissue damage due to the combination of a yet shorter path length (50–80 µm) combined with a higher linear energy transfer (100 KeV·µm^−1^). This results in far greater cytotoxicity per particle traversal of the cell nucleus [11,12].

High LET α-particle radiation depresses enzyme repair mechanisms (RAD51), impacts radiosensitivity during cell cycle, delays in cell division, and induces apoptosis thereby decreasing the protective effects of neighboring cells in organized systems [13,14,15]. This complexity, leading to increased chromosome aberrations compromises the DNA repair process from high-LET damage, making it more difficult for cells to repair double strand break damage and survive as compared with low-LET damage. Studies from this laboratory using a peritoneal xenograft model (LS-174T) have shown that ^212^Pb-labeled trastuzumab-induced cell death in the tumor occurs principally by G2 arrest, accompanied by a delay in DNA damage repair linked to the down regulation of RAD51 and thus a compromised ability to respond to and repair the hallmark double strand break damage of high-LET radiation, and modulation of genes after exposure to a therapeutic dose of ^212^Pb [13,14].

Execution of a study with the β^−^-emitting ^177^Lu-trastuzumab RIT (low-LET therapy) was performed to directly compare the results to those obtained from the corresponding α-emitting (high-LET therapy) ^212^Pb-trastuzumab therapy, using the identical *in vivo* tumor model conditions at their respective optimal therapy doses. A parallel objective was to obtain the control study for high-LET RIT that is lacking in the literature. Additionally, looking forward, a regimen combining ^177^Lu and ^212^Pb for the treatment of tumor and lesions of varying sizes and volumes might provide an enhanced RIT therapy. Combining these two radionuclide generated particles has been demonstrated to be potentially beneficial for cancer therapy [15]. Therefore, the aim of the present study was to bring to light the mechanisms of cell killing induced by ^177^Lu-trastuzumab and to directly compare these results with the prior data obtained from ^212^Pb-trastuzumab [13,14]. Comparison of the results allows: (1) a better definition of the differences in *in vivo* tumor cell killing between low- and high-LET radiation; and (2) represent a starting point for future clinical investigations of combination α- and β^−^-radiation therapies for intraperitoneal disease. Clinical translation of these studies would greatly expand the cross section of patients that might benefit from HER2 targeted therapies. The studies reported herein describe the various biological responses such as apoptosis, cell cycle distribution, DNA repair, metaphase spread, and gene modulation involved in apoptosis in the xenograft tumors that were treated with ^177^Lu-RIT.

## 2. Results

### 2.1. ^177^Lu-Trastzumab Induces Apoptosis in Intraperitoneal (i.p.) Human Colon Carcinoma Treated Xenografts

**Figure 1:**
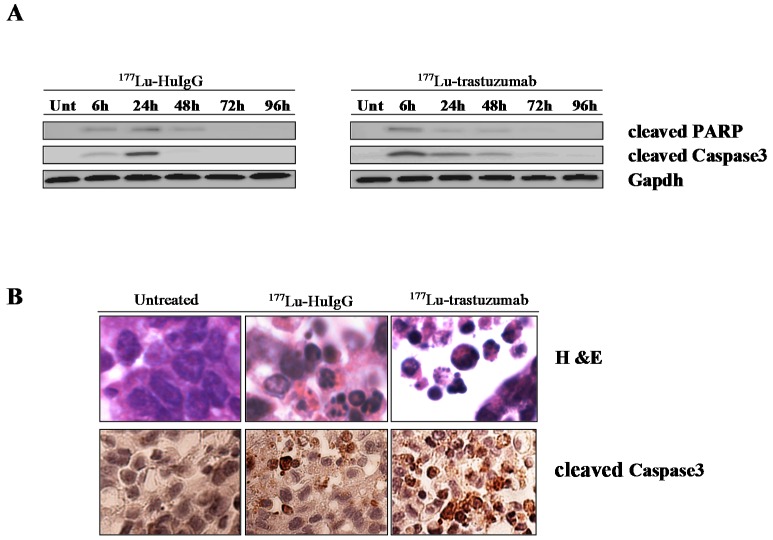
Induction of apoptosis in human colon cancer LS-174T intraperitoneal (i.p.) xenografts following ^177^Lu-trastuzumab treatment. (**A**) Tumor bearing mice were treated with ^177^Lu-trastuzumab and the tumor tissue was collected at the indicated times. Additional groups included ^177^Lu-HuIgG as a non-specific control and untreated mice. Immunoblot analysis for caspase-3 and PARP was conducted and both cleaved caspase-3 (17 kDa) and PARP (89 kDa) were observed. The equivalent protein loading control was GAPDH; (**B**) Apoptosis induced by ^177^Lu-trastuzumab. Tumor bearing mice were treated with ^177^Lu-trastuzumab. Additional groups included ^177^Lu-HuIgG as a non-specific control and untreated mice. Paraffin-embedded sections were stained with haamatoxylin and eosin (H & E) or cleaved caspase-3 antibody under light microscopy (40× magnification).

^177^Lu-trastuzumab was administered to mice bearing i.p. xenografts (LS-174T). To examine the role of caspase activation in β^−^-particle irradiation-induced apoptosis, the expression of cleaved caspase-3 and PARP was assessed using immunoblot techniques. Caspase-3 mediates the nuclear enzyme PARP through proteolytic cleavage, which then plays an important role in apoptotic cells. Thus, the caspase-3 and PARP are established and reliable indicators of apoptosis. Treatment with either ^177^Lu-trastuzumab or ^177^Lu-HuIgG resulted in caspase-3 and PARP activation (Figure 1A). The difference between the specific and non-specific ^177^Lu-RIT is evident in the intensity of that response at 6 h following the administration of the ^177^Lu-trastuzumab. In tumor treated with the non-specific control, ^177^Lu-HuIgG, PARP and caspase-3 are more pronounced at a later time point (24 h) and the intensity of caspase-3 is markedly reduced at 48 h. On the contrary, the intensity of cleaved caspase-3 in tumor treated with the specific ^177^Lu-trastuzumab is more evident at 48 h. The activation of caspase-3 and the presence of apoptotic bodies also exhibited clear differences in apoptosis (Figure 1B) *versus* the ^177^Lu-HuIgG treated tumor as depicted by immunohistochemistry (IHC) and haamatoxylin and eosin (H & E) staining, indicating effective, specific, targeted tumor killing by ^177^Lu-trastuzumab.

### 2.2. ^177^Lu-Trastuzumab Induces DNA Damage and Interferes with DNA Repair

**Figure 2:**
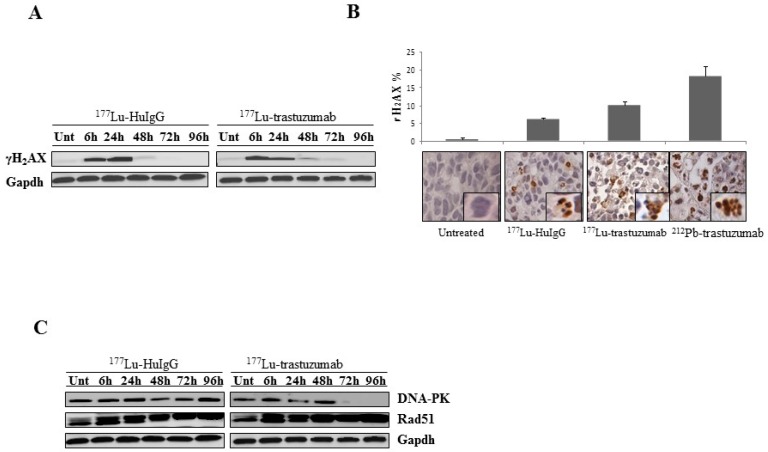
^177^Lu-trastuzumab-induced DNA damage in human colon cancer LS-174T i.p. xenografts. (**A**) Tumor bearing mice were treated with ^177^Lu-trastuzumab as indicated and the tumor was collected for 96 h. ^177^Lu-HuIgG was included as a non-specific control along with untreated mice. Western blot analysis for γH_2_AX was conducted and detected at 17 kDa. GAPDH was the equivalent protein loading control; (**B**) Light microscopy image of untreated control, ^177^Lu-HuIgG, ^177^Lu-trastuzumab, and ^212^Pb-trastuzumab. γH_2_AX was clearly identified on IHC staining compared to the untreated control in tumor tissue 24 h post RIT (40× magnification). The percentage of cells staining positive for γH_2_AX were plotted. Results represent an average of at least three replications; (**C**) Expression of repair proteins induced by ^177^Lu-trastuzumab. Xenograft mice (i.p. LS-174T) were treated with ^177^Lu-trastuzumab. Additional groups included untreated and ^177^Lu-HuIgG as a non-specific control. Immunoblot analysis for DNA-PK and Rad51 were performed. DNA-PK was detected at 450 kDa. Rad51 was detected at 37 kDa. The equivalent protein loading control was GAPDH.

The findings detailed above suggest that the cytotoxic effect of ^177^Lu-trastuzumab in the targeted tumor causes DNA double strand breaks (DSBs), perhaps the most harmful cytotoxic damage for a cell. As such, the induction of DNA-damage in the human colon carcinoma xenografts by ^177^Lu-trastuzumab was investigated. Immunoblotting was performed with tumors harvested at the designated time points to detect γH_2_AX, a marker for DNA-double strand damage (Figure 2A). An intense increase in γH_2_AX from ^177^Lu-trastuzumab treated tumor is evident starting at 6 h and is down-regulated in a time dependent manner. Meanwhile, in tumor treated with the non-specific control, ^177^Lu-HuIgG, γH_2_AX was more pronounced at a later time point (24 h). However, as with the previous study, γH_2_AX is still present at 48 and 72 h in the ^177^Lu-trastuzumab treated tumors while the signal is markedly reduced after 48 h in the tumors exposed to the ^177^Lu-HuIgG.

The increase in γH_2_AX was also confirmed and quantitated by IHC using paraffin-embedded tumor sections. Again, a significant induction of DNA damage was observed in tumors that were treated with ^177^Lu-trastuzumab in which 10.2% of the cells are positive for γH_2_AX as shown in Figure 2B. Tumors treated with ^177^Lu-HuIgG also had a population of cells (6.7%) that were positive following treatment. The difference (1.5-fold) between the specific and non-specific targeting vectors suggests a specific effect due to the ^177^Lu-trastuzumab. However, when compared to tumors treated with ^212^Pb-trastuzumab, the intensity and percentage of cells (18.5%) that stained positive for γH_2_AX was 1.8-fold greater than the ^177^Lu-trastuzumab treated tumors. This suggests that DSB damage resulted more efficiently following exposure to high-LET radiation and/or that the apparent DSB detected from the low-LET radiation was the result of a very large number of single strand breaks that effectively equate to DSBs.

To identify DNA repair pathways involved with ^177^Lu-trastuzumab in these repair mechanisms, DNA-PK and RAD51, which play important roles in non-homologous end joining (NHEJ) and homologous repair (HR), respectively, were investigated using immunoblotting methods. As illustrated in Figure 2C, the interference of DNA-PK in both specific and non-specific ^177^Lu-RIT is present at the earlier time points at 24 and 48 h, respectively. However, down-regulation of DNA-PK following treatment with ^177^Lu-trastuzumab was most evident at the later time points (72 and 96 h) while DNA-PK was present throughout the 96 h period following treatment with the control ^177^Lu-HuIgG. Therefore, inhibition of DNA damage repair induced by ^177^Lu-trastuzumab treatment, evidenced by the reduction of DNA-PK protein expression, may be an explanation for the increased cell killing efficacy of ^177^Lu-trastuzumab treatment.

Conversely to what was observed for treatment with ^212^Pb-trastuzumab, Rad51 expression was not affected in the presence of ^177^Lu-trastuzumab and ^177^Lu-HuIgG at the indicated time points.

### 2.3. Metaphase Spread

**Figure 3:**
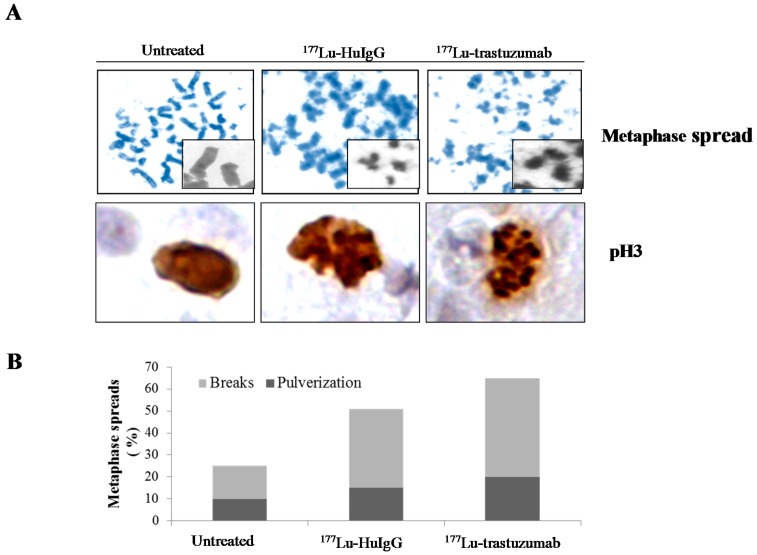
^177^Lu-trastuzumab-induces chromosomal abnormality in human colon cancer LS-174T i.p. xenografts. (**A**) Representative images of mitotic chromosomes from tumors that received ^177^Lu-trastuzumab from tumor bearing mice that had been treated with ^177^Lu-trastuzumab. Additional groups of animals included untreated and those treated with ^177^Lu-HuIgG (non-specific control). Paraffin-embedded sections were stained (H & E and pH 3 antibody) under light microscopy (40× magnification); (**B**) Metaphase spreads with chromosome breaks percentage and/or pulverized aberrations in tumor cells that received ^177^Lu-trastuzumab. Results represent an average of a minimum of three replications.

To examine the effect of ^177^Lu-RIT on mitotic entry, chromosomal spreads were prepared from tumors treated with ^177^Lu-trastuzumab. Treatment with ^177^Lu-trastuzumab resulted in sister chromatid cohesion failure and with an accumulation of chromosomal breaks (Figure 3A). Metaphase spreads from tumors treated with ^177^Lu-trastuzumab showed predominantly chromosomal breaks and pulverization. The average number of chromosomal breaks per metaphase was greater in tumors treated with ^177^Lu-trastuzumab than those with ^177^Lu-HuIgG, 63% *versus* 50% (*p* < 0.05), respectively (Figure 3B), indicating that ^177^Lu-trastuzumab specifically induces DNA damage and blocked chromatin cohesion.

Phosphorylated Histone H3 (pH 3), an indicator of mitotic entry, was also examined using IHC. Compared to the untreated control, tumor tissue from the ^177^Lu-trastuzumab treatment group demonstrated an elevated induction of pH 3. There was also an associated abnormal morphology, a general characteristic of apoptosis, along with the fragmentation of the nuclei into several bodies accompanied by condensation of nuclei into dense particles (Figure 3A). Furthermore, this effect was more pronounced in the group treated with the ^177^Lu-trastuzumab than the control ^177^Lu-HuIgG.

### 2.4. ^177^Lu-Trastuzumab Attenuates Proliferation in S Phase and Induces G2 Cell Cycle Arrest

**Figure 4:**
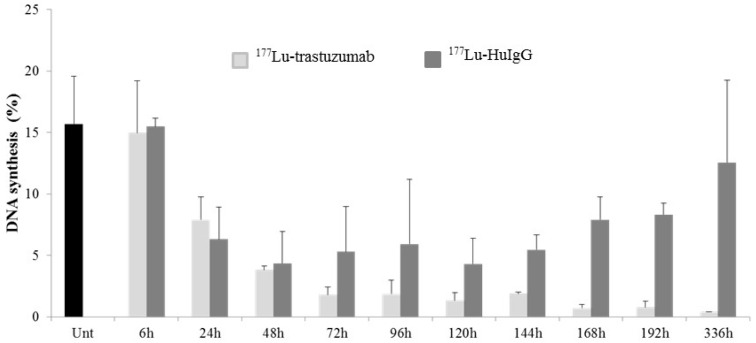
Analysis of DNA synthesis in LS-174T tumor xenografts following treatment with ^177^Lu-trastuzumab. Values are the average and standard deviation of three analyses.

The effect of ^177^Lu-RIT on cell cycle distribution was assayed. In this study, mice bearing i.p. tumor xenografts were treated with either ^177^Lu-labeled trastuzumab or HuIgG and then administered BrdU 4 h prior to tumor harvesting to pulse-label the DNA. DNA synthesis and the cell cycle of the treated tumors were then evaluated using flow cytometry. Tumor cells harvested from untreated mice were found to have the expected uptake of BrdU (15.8%); the resulting cell cycle distribution was also in the expected range. As shown in Figure 4, by 24 h after administration of ^177^Lu-trastuzumab, there is a noticeable decrease in BrdU incorporation (7.9%). The incorporation decreased further to 3.8% at 48 h and remained at this low level throughout the remainder of the 336 h period; re-initiation of DNA synthesis was not evident within this time frame in the treated group. The non-specific control, ^177^Lu-HuIgG, also elicited a cessation of DNA synthesis until 48 h. In contrast to the ^177^Lu-trastuzumab results, DNA synthesis does re-initiate at 72 and 96 h (^177^Lu-HuIgG at 48 h *vs.* ^177^Lu-HuIgG at 96 h, *p* < 0.05) after treatment with ^177^Lu-HuIgG, indicating that the continued cessation of DNA synthesis is specific to the β^−^-particle ^177^Lu-trastuzumab-RIT (Figure 4).

In addition, treatment with ^177^Lu-trastuzumab or ^177^Lu-HuIgG, the S phase fraction of the cell cycle decreased with a corresponding increase in G2/M phase beginning at 48 h in the targeted tumors when compared to tumors collected from untreated mice (untreated *vs.* ^177^Lu-trastuzumab, *p* < 0.05; untreated *vs.* ^177^Lu-HuIgG, *p* < 0.05). The decrease in S phase and elevation of the G2/M phase fractions were maintained for 336 h study period in the tumors treated with ^177^Lu-trastuzumab (untreated *vs.* ^177^Lu-trastuzumab, *p* < 0.001; untreated *vs.* ^177^Lu-HuIgG, *p* < 0.05). Meanwhile, by 168 h, S phase distribution percentage had rebounded in those tumors collected from mice treated with non-specific control ^177^Lu-HuIgG (untreated *vs.* ^177^Lu-HuIgG, *p* < 0.05). Continued depression of S phase and elevation of G2/M fractions beyond 168 h appears specific to ^177^Lu-trastuzumab treatment (untreated *vs.* ^177^Lu-trastuzumab, *p* < 0.001) (Table 1).

After each treatment, the mice (*n* = 5) used for DNA synthesis were injected with BrdU 4 h prior to euthanasia. All results here represent an average of a minimum of three replicates (±S.D.).

### 2.5. Up-Regulation of Genes Involved in Apoptosis Is Delayed by ^177^Lu-Trastuzumab Therapy

To further investigate the mechanistic basis of β^−^-particle RIT from ^177^Lu-trastuzumab, gene expression was analyzed using Polymerize Chain Reaction (PCR) array in three additional experiments. The PCR array identified significantly up- or down-regulated genes 24 or 168 h after ^177^Lu-trastuzumab therapy (Table 2). All experimental group results were compared to untreated tumor as a control with application of 2-fold change threshold.

Eighty-four genes were examined (Table S1), of which thirteen genes involved in apoptotic regulation processes were assayed in the PCR array and alterations in the expression of those genes in the treated tumor xenografts are presented in Table 2. Ten of those genes were up- or down-regulated in tumor tissues collected that had been treated with either ^177^Lu-trastuzumab or ^177^Lu-HuIgG. Three of those genes fell below the 2-fold cut off. No significant changes were observed at 24 h in the tumors treated with ^177^Lu-trastuzumab except for *BRCA1* (2.9-fold decrease, *p* < 0.013) and *CIDEA* (7.0-fold decrease, *p* < 0.001), the latter demonstrating the largest change. Furthermore, *CIDEA* was the only gene for which there was a notable difference between the specifically (trastuzumab) and non-specifically (HuIgG) targeted ^177^Lu-RIT (*p* < 0.01).

In contrast, at the 168 h time point, the expression of eight genes showed a significant change in expression. Up-regulation was noted for six genes while two were down-regulated. Furthermore, five genes continued over time in the same directional change of expression. That is, *BRCA1* and *RAD21* showed a further decrease in their expression while *GADD45*, *GADD45γ* and *PCBP4* increased, albeit modestly. More noteworthy was that six genes that had decreased in expression, converted to an increase of expression at 168 h following ^177^Lu-RIT. Four of these genes (*ABL*, *CIDEA*, *GML* and *IP6K3*) also showed a substantial difference in expression between those tumors collected from the ^177^Lu-trastuzumab and those from the ^177^Lu-HuIgG treated groups.

### 2.6. ^177^Lu-Trastuzumab-Induced Tumor Cytotoxicity May Be Associated with Differential Expression of Genes Involved in Cell Cycle Arrest and Cell Cycle Check Point Regulation

Of the 84 genes examined (Table S1), those genes involved cell cycle arrest (15 genes) and cell cycle checkpoint (eight genes) regulation were represented in the PCR array used in this profiling study. Twelve of those genes were significantly modulated in tumor tissues harvested from specific or non-specific treated tumor bearing animals. The rest of the genes involved in these categories fell below the 2-fold cut off.

Only one gene (*SESN1*) was found to be up-regulated while four genes (*BRACA1*, *DDIT3*, *FANCG* and *MAPK12*) were down-regulated 24 h after ^177^Lu-trastuzumab or ^177^Lu-HuIgG treatment (Table 3). *BRACA1* and *FANCG* are involved in the regulation of the cell cycle checkpoint while the other three identified genes have roles in cell cycle arrest. At 24 h, the ^177^Lu-trastuzumab treatment elicited no significant differences as compared to the non-specific control group (*p* > 0.05).

One week after exposure to ^177^Lu-RIT, among those genes involved with cell cycle arrest, *CHK1*, *CHK2*, *GML*, and *GTSE1* showed the greatest differences in their expression. The expression of *CHK1*, *CHK2*, and *GTSE1* decreased further from 24 h while *GML* transitioned from a 1.6-fold decrease in expression to a 5-fold increase. The greatest effect was observed in the *GTSE1* with a 9.0-fold decrease in expression. There was also a clear difference in those genes compared to non-specific control (*CHK2*, *GTSE*; *p* < 0.01, *GML*; *p* < 0.05).

Of the eight genes associated with the cell cycle checkpoint, only alterations in *BRCA1*, *FANCG* and *NBN* expression were notable at either 24 or 168 h. *BRCA1* and *FANCG* demonstrated further suppression of expression at 168 h following ^177^Lu-RIT. In this category of genes, the greatest impact was observed with *BRCA1* with a 2.9-fold and 6.6-fold decrease at 24 and 168 h, respectively. Significant differences between the ^177^Lu-trastuzumab and ^177^Lu-HuIgG treated tumor tissue were observed (*p* < 0.05).

### 2.7. Expression of Genes Involved in Repair of Damaged DNA Induced by ^177^Lu-Trastuzumab

This PCR array was also utilized to delineate DNA repair genes involved in response to ^177^Lu-trastuzumab treatment. The genes probed include those involved in repair of single strand breaks of DNA (SSB), nucleotide excision repair (NER; 12 genes), base-excision (BER; seven genes), mismatch repair (MMR; 14 genes) and repair of double-strand breaks (DSB; nine genes). Again, applying an exclusion requirement of a 2-fold difference, a total of eight genes were recorded with expression changes at 24 h after ^177^Lu-RIT treatment (Table 4). Three of the genes (*BTG2*, *MRE11A*, and *XPC*) were upregulated and five genes (*BRCA1*, *FANCG*, *PNKP*, *RAD18*, and *UNG*) were downregulated. However, only two genes (*BTG2* and *MRE11A*) demonstrated a clear difference between the specific and non-specific ^177^Lu-RIT treatment (*p* < 0.05). In fact, treatment of mice with ^177^Lu-HuIgG resulted in a higher tumor expression of *BTG2* than did the ^177^Lu-trastuzumab. *BTG2* falls into a general category of genes that are related to the repair of DNA while *MRE1A* is involved in the repair of DSBs.

In contrast, at 168 h after ^177^Lu-RIT, twelve genes were identified that either exhibited increases (three) or decreases (nine) in expression. Among the downregulated genes (*BRCA1*, *EXO1*, *FEN1*, *MSH2*, *NBN*, *PRKDC*, and *RAD51B*), a statistical difference between the ^177^Lu-trastuzumab and ^177^Lu-HuIgG was found for all but *EXO1* (*p* < 0.05). *BTG2*, *p73*, and *XPC* were up-regulated, however the differences in these between ^177^Lu-trastuzumab and ^177^Lu-HuIgG treated tumor tissue was not significant. *BRCA1*, demonstrated the greatest change in expression from 24 to 168 h, 2.9-fold to 6.6-fold, respectively. The genes exhibiting the greatest level of change are involved in DDB (five genes), MMR (one gene) and DSBR (one gene). When the entire list of responsive genes is examined, the only gene not represented as a repair mechanism invoked by ^177^Lu-RIT was *BER*.

## 3. Discussion

A critical feature of radiation damage originated from delivery of ionizations or energy deposition directly in, or at the cell nucleus, or very close to the DNA. The resulting molecular damage includes a host of possible single or double strand breaks, base damage, cross-links and other compromising damage to the DNA. The deposition of energy in tissue is different between low-LET γ- or β^−^-irradiation and high-LET α-particle irradiation. Cellular effects originating from high-LET radiation are dominated by a greater impact and therefore result in far less repairable damage. These clusters of damage are expected to be qualitatively different from low-LET radiation damage [13,16,17]. With these previous observations in mind, tumor bearing mice (i.p. LS-174T xenografts) were treated with low-LET radiation, specifically, ^177^Lu-labeled trastuzumab. The data gathered from these studies can then be directly compared to the previously published data on the molecular effects obtained using the same tumor model system treated with α-particle high-LET radiation using ^212^Pb with respect to cell killing mechanisms *in vivo* [13,14].

Anticancer drugs, β^−^-radiation and γ-irradiation have been shown to activate apoptosis pathways in leukemias and solid tumors [18,19]. Figure 1 shows fragmentation of the nuclei typically observed in apoptosis. Caspases play a critical role in apoptosis induction [20,21]. The effect of ^177^Lu-trastuzumab on apoptosis in human colon cancer LS174T i.p. xenografts appears to be more pronounced at the earliest time point (6 h) assessed compared to the non-specific ^177^Lu-HuIgG control as evidenced by expression of cleaved caspase-3 (Figure 1). Just as important is that this response persists out to 72 h whereas cleaved caspase-3 exhibits high response at a later time point (24 h) and is no longer present at 48 and 72 h in the ^177^Lu-HuIgG treated tumors. These observations indicate that ^177^Lu-trastuzumab induces apoptosis via caspase-3 dependent mechanisms. In contrast, caspase-3 was not activated by ^212^Pb-trastuzumab treatment [13], suggesting that α- and β^−^-particle RIT may operate through different cell killing mechanisms. Taking into account the complexity of the *in vivo* environment, it is also possible that the magnitude of the molecular evaluations and assays results begin to approach the limit of detectability levels making recognition of some of molecular differences harder to fully appreciate and even discern. Nevertheless, it does remain clear that the β^−^-particle RIT results in measureable differences at the molecular level *versus* controls and those same detected differences may in part explain the overarching therapy results that have been reported using this tumor model system. A relatively high expression of caspase-3 in tumor tissues was noted at a later time point (24 h) in the control group implicating that other factors, such as the bystander effect and/or cross-fire, which may indeed be of some importance. Deconvoluting these effects and mechanisms would be suitable future studies that we would pursue. The non-specific effect therapy and bystander effects in α-RIT have been well recognized and acknowledged in the past and are not unexpected with β^−^-RIT [13,22]. However, we are not aware that β^−^-particle RIT has higher bystander effect than α-particle RIT. Therefore, consideration toward greater and more specifically detailed studies of these biological responses (bystander) and the physical effects (cross-fire) must be performed in the future.

To determine the effect of β^−^-particle RIT on DNA synthesis, mice were injected with BrdU to pulse-label the i.p. tumor xenografts. Decreased levels of DNA synthesis occurred at early time points in the ^177^Lu treated tumor and persisted throughout the study period (336 h) for the ^177^Lu-trastuzumab treated tumors, but appeared to rebound and resume synthesis at 72 and 96 h in the ^177^Lu-HuIgG treated tumors (Figure 4). By the end of the study period, DNA synthesis in the control treated groups nearly returned to levels found in tumors from the untreated mice. This pattern is very similar for tumor treated with ^212^Pb-trastuzumab, although the recovery of DNA synthesis is not apparent, however in those studies due to half-life considerations that study was followed for a shorter period of time [13]. Differences are apparent in tumors from the non-specific control. The DNA synthesis of tumors taken from mice treated with ^212^Pb-HuIgG showed signs of DNA synthesis resumption by 120 h.

Specifically, treated tumor cells harvested from xenograft bearing mice remained arrested at the G2/M phase with the S phase depressed beyond 168 h. Synchronization in the G2/M phase after 168 h treatment combined with persistent reduction of DNA synthesis seems the most prominent difference between the targeted ^177^Lu-trastuzumab and the non-specific control, ^177^Lu-HuIgG (Table 1). The majority of cancer cells were deficient in the G1-S DNA damage checkpoint (due to p53 mutation). This leads to a reliance on the S and G2 checkpoints for possible DNA repair and survival. This temporal progression was observed for the phase distribution of cells following ^212^Pb-RIT [13]. Tumor cells were arrested in the G2/M phase with a severely depressed S phase after the 24 h time point and appeared to rebound in the tumors from ^212^Pb-HuIgG treated mice after 120 h, but not for ^212^Pb-trastuzumab. The lower S phase and the elevated G2/M phase fractions in tumors from mice given ^212^Pb-trastuzumab were more prominent at 6 and 24 h, compared with ^177^Lu-trastuzumab.

One of the most important types of radiation damage in DNA is double stranded breaks. γH_2_AX facilitates an assembly of damage responsive proteins and chromatin remodeling complexes to those locations associated with DNA damage and consequently influences the accuracy and efficiency of DNA repair [23,24,25]. Although β^−^-particle radiation using ^117^Lu-RIT induced DNA damage and exhibited apoptotic characteristics as expected, more severe DNA damage was observed in tumors treated with ^212^Pb-trastuzumab (Figure 2). HR and NHEJ are the predominant mechanisms in DSB repair involving Rad51 and DNA-PK, respectively. The absence or down-regulation of either mechanism results in the failure to repair that form of damaged DNA, respectively, giving rise to mutations, chromosomal abnormalities, and reproductive cell death. The degree of misjoining frequencies has been demonstrated to be greater originating from high-LET radiation than that from low-LET radiation [26,27,28]. Interestingly, differences for the NHEJ system (DNA-PK) were evident at later time points for ^177^Lu-trastuzumab treatment while this indicator was not affected by ^212^Pb-trastuzumab treatment [13]. The results reported herein suggest that interference with the NHEJ repair systems occurs in the tumors in response to treatment with ^177^Lu-trastuzumab, while conversely when treated with ^212^Pb-trastuzumab the HR repair systems, including Rad51, were effectively taken off-line compromising response and repair. Thus, it appears that the specific repair mechanism most directly related to the specific form of radiation damage, low- *vs.* high-LET in origin, was negatively impacted leading to efficient tumor cell killing with both forms of radiation.

The gene profiling study indicated that Rad51B, a Rad51 homolog, was also down-regulated in tumors one week (168 h) after injection mice with ^177^Lu-trastuzumab. Ultimately, adequate numbers of SSBs may effectively equate to substantial DSBs resulting in cell death from low-LET radiation traversal of tumor cell nuclei. Based on this result, suppression of HR repair may also be invoked by low-LET radiation leading to tumor cell death. Down-regulation of Rad51, DNA-PK, or Ligase IV leads to a significant increase in fragile site expression under replication stress. The repair of these breaks is essential for chromosomal stability. Indeed, chromosomal breaks and sister chromatid cohesion failure were noted in tumors specifically treated with β^—^RIT, suggesting that cells in such treated tumors may enter mitosis, but then also do so with accumulated chromosomal abnormalities that consequently lead to the death of those treated cells (Figure 3).

A critical biological process to guard against damaged and mutated cells in response to DNA damage is the activation of programed cell death. Radiation exposure can lead to complicated cellular responses including alteration in gene expression. Recently, Schuler *et al.* demonstrated that metabolic and stress response related transcripts were most prevalent after exposure to ^177^Lu in mouse kidney tissue [29]. The gene profiling study presented here revealed differentially expressed genes after treatment with ^177^Lu-trastuzumab. Six genes (*ABL*, *GADD45α*, *GADD45γ*, *GML*, *IP6K3*, and *p73*) involved in apoptosis were found to be up-regulated at 168 h in tumors treated with ^177^Lu-trastuzumab. Although *GADD45*, *GML*, *IP6K3*, and *PCBP4* have been known to induce *p53* mediated apoptosis, *p53* activation itself was not observed resultant from ^177^Lu-trastuzumab treatment. Thus, induction of genes involved in cell death by ^177^Lu-trastuzumab treatment appears independent of *p53*. *p73* is one of the *p53* tumor suppressor family and is involved in cell cycle arrest and induction of cell death due to DNA damage and there are tumors that lack a functional *p53* that do then functionally express *p73* [29,30,31,32]. Interestingly, upregulation of gene expressions by ^177^Lu-trastuzumab treatment at 168 h, albeit a much lesser expression, was similar to those observed from ^212^Pb-RIT at 24 h [13]. Activation of *p73* might then be involved in the initiation of apoptosis in response to ^177^Lu-trastuzumab. The up-regulated genes by ^177^Lu-trastuzumab therapy could lead to induction of apoptosis, possibly as a result of activation of *GADD45/p73* signaling. Studies from this laboratory have previously reported that *p38* mediated activation of *p73* may be active in apoptosis initiation, as evidenced by those genes expressed in response to ^212^Pb-trastuzumab [13]. Increased expression of *GML* and *IP6K3* was not detectable at 24 h following the exposure to β^−^-particle RIT while significant modulation of those genes was observed later at 168 h. Increased expression of *GML* and *IP6K3* may be a specific response to ^177^Lu-trastuzumab. *GML* has been implicated in the enhancement of G2/M arrest and apoptosis following γ-irradiation [33].

The gene profile study identified a total of five genes at 24 h and nine genes at 168 h related to cell cycle that were found to be differentially impacted in the ^177^Lu-RIT treated xenografts. Differences between specific and non-specific targeted ^177^Lu-RIT were negligible. At 168 h, however, significant differences in expression between the two groups were found for *BRCA1*, *CHK1*, *CHK2*, *GML*, *GTSE1*, and *NBN.* *BRCA1* deficiency results in S-phase checkpoint abnormalities, the G2/M checkpoint, centrosome duplication and spindle checkpoints. *CHK2* (Chk2 checkpoint homolog) is activated after radiation exposure. *CHK2* activity is also required for phosphorylation of the dual specificity phosphatases Cdc25A/C, which inactivates those enzymes, thereby causing a G2/M arrest by blocking CDK1 activation. CHK2 also phosphorylates BRCA1, which permits BRCA1 to restore survival after DNA damage [34,35]. *GTSE1* is expressed only in the S and G2 phases. *GTSE1* binds p53 in the nucleus in response to DNA damage shuttling it out of the nucleus thereby compromising its ability to induce apoptosis [36]. Compared to mice treated with ^212^Pb-trastuzumab, *DDIT3* and *SESN1* were the only cell cycle related genes that were differentially modulated at 24 h in mice specifically treated with ^177^Lu-trastuzumab. Interestingly, the six genes (*BRCA1*, *CHK1*, *CHK2*, *GML*, *GTSE1*, and *NBN*) modulated 168 h after ^177^Lu-trastuzumab treatment were not identified in the same mouse model at 24 h after exposure to ^212^Pb-trastuzumab. Therefore, these results may be a specific response by the cells in response to ^177^Lu-trastuzumab.

DNA damage potentially compromises the integrity and translation of critical information in the genome. Improper DNA repair and corruption can cause an array of problems that can result in death of the cell, including mutation, chromosome aberration, genetic instability, and oncogenic transformation. Direct comparison here of the expression of DNA damage repair genes revealed increased gene modulation in *BRCA1*, *EXO1*, *FEN1*, *MSH2*, *NBN*, *PRKDC*, *RAD21*, *RAD51B*, and *p73* at 168 h after treatment with ^177^Lu-trastuzumab compared to the non-specific control. Alterations in the expression of those genes were modest at 24 h following the exposure to β^−^-particle RIT while significant modulation of those genes was observed at 168 h. Among those genes, *EXO1*, *MSH2*, and *p73* falls into MMR while *FEN1*, *NBN*, *PRKDC* and *RAD21* are involved in DSBR, suggesting that ^177^Lu-trastuzumab compromises repair of both single and double strand breaks at a later time, especially with the coordination of MMR and DSDB. MSH2 is involved in many different forms of DNA repair that are associated with some cancers [37]. The non-homologous end joining (NHEJ) pathway of DNA repair which responds to DSBs requires PRKDC [38]. Notably, the pattern of gene modulation in the DNA repair category was quite different for tumors treated with ^212^Pb-trastuzumab, where only three genes *(BTG2*, *ERCC1*, and *XPC*) of DNA repair was the same as those observed at 24 h after exposure to ^177^Lu-trastuzumab. Negligible effects in gene modulation were discerned between the tumors from the mice administered ^177^Lu-trastuzumab and ^177^Lu-HuIgG. Genes (*EXO1*, *FEN1*, *MSH2*, *NBN*, *PRKDC*, *RAD21*, and *RAD51B*) found affected in the mice 168 h after exposure to ^177^Lu-trastuzumab were not observed in the mice treated with ^212^Pb-trastuzumab, indicating that those genes may again be specific to β^−^-RIT. This should be unsurprising considering that apoptosis, cell cycle arrest and DNA damage repair were impacted at later time points in tumors treated with ^177^Lu-RIT compared to ^212^Pb-RIT. This result suggests that different mechanisms may be involved in the cell killing effected by α- or β^−^-emitting radionuclides.

## 4. Materials and Methods

### 4.1. Cell Line

To maintain direct comparability to prior studies, LS-174T, a human colon carcinoma cell line was used and grown in supplemented Dulbecco’s Modified Eagle’s Medium (DMEM) exactly as previously described with all media and supplements being procured from Lonza (Walkersville, MD, USA) [39]. Mycoplasma and other pathogen screening were executed prior to *in vivo* use in accordance to the policies of the National Cancer Institute (NCI) Laboratory Animal Sciences Program without any further cell line authentication.

### 4.2. Preparation of ^177^Lu-Trastuzumab and ^177^Lu-HuIgG

The bifunctional acyclic CHX-A″-DTPA chelating agent was conjugated to trastuzumab using established methods (10-fold molar excess of ligand to trastuzumab reaction ratio) as previously reported [4,40]. The concentration of the protein of the immunoconjugate was determined (Lowry) and the average number of chelates per mAb assayed by a literature spectrophotometric method [41,42]. Lutetium-177 (110 mCi) (PerkinElmer, Shelton, CT, USA), dissolved in 100 µL of 0.1 N HCl, was adjusted to pH 5.5 (5 M NH_4_OAc buffer (pH 5.5)). The CHX-A″-trastuzumab immunoconjugate (2.0 mg) in 0.15 M NH_4_OAc buffer (pH 6.5–7.0) was added to the buffered ^177^Lu. After incubation (1 h, 37 °C), the radiolabeling reaction was halted with 0.1 M EDTA (4 µL, pH 6.0). Purification of the radioimmunoconjugate (RIC) was then affected by use of a PD-10 desalting column (GE Healthcare, Piscataway, NJ, USA). The non-specific control antibody for these experiments was prepared from HuIgG (MP Biomedicals, Santa Ana, CA, USA) using the identical conditions described for the specific agent.

### 4.3. Tumor Model, Treatment, and Tumor Harvesting

Animal protocols were approved by the National Cancer Institute Animal Care and Use Committee for all experiments (ROB-104 & ROB-105). Trypan-blue was used to determine the viability of the LS-174T cells (>95%). Studies were performed with 19–21 g female athymic mice (The Frederick National Laboratory for Cancer Research) injected i.p. with 1 × 10^8^ LS-174T cells in 1 mL of DMEM. This specific inoculum provides the minimum number of cells required to generate 100% xenograft growth in the animals generating the identical model system of 3-day intraperitoneal (i.p.) LS-174T xenografts as previously reported [43]. ^177^Lu-trastuzumab (375 µCi in 0.5 mL PBS) was administered i.p. to these mice. Tumors were harvested thereafter at the listed time points (*n* = 10–15 per time point). Control groups of mice received the control ^177^Lu-HuIgG, or no treatment. The amount of tumor collected was not measured; however, at Day 7, the tumor burden is typically 128.5 ± 205.6 mg.

Mice were euthanized by CO_2_ inhalation at a flow rate of 2 L/min. When breathing ceased for all mice, tumor tissue was harvested from the peritoneum. Tumor tissue collected at each time point, pooled, visually inspected, and adherent tissues removed. The tumor tissue was then completely washed with ice-cold PBS 3 times, divided, and processed as required for each assay. Mice (*n* = 3–5) utilized for the cell cycle and DNA synthesis studies were injected i.p. with 5-bromo-2′-dexoyuridine (BrdU; 1.5 mg in 0.5 mL PBS; Sigma, St. Louis, MO, USA) 4 h prior to euthanasia. The tumors were collected, fixed and stored for flow cytometry analysis as previously described [13].

### 4.4. Flow Cytometry Measurements

DNA synthesis and cell cycle distribution were assayed by flow cytometry as previously described with some modifications [13,44]. The ethanol-fixed tumor tissue was washed in cold PBS and incubated in 1 mL 0.04% pepsin (*w*/*v*; Sigma) in 0.1 N HCl (Mallinckrodt, Inc., St Louis, MO, USA). After 1 h at 37 °C with shaking, the digest was centrifuged and the pellet re-suspended in 2 N HCl (1 mL; Mallinckrodt, Inc.), and then incubated at 37 °C for 20 min with shaking. The nuclear suspension was neutralized with 0.1 M sodium tetraborate (Sigma), washed with phosphate buffered saline containing 0.5% bovine serum albumin and 0.5% Tween-20 (PBTB) and re-suspended in PBTB. The nuclei (100 µL) were incubated with 20 µL of FITC-labeled anti-BrdU mAb (BD Biosciences, San Jose, CA, USA) for 1 h at 4 °C, washed in cold PBS and re-suspended in 2 mL of propidium iodide solution (50 µg/mL in PBTB; Sigma) containing RNAse A (50 µg; Sigma) and incubated for 30 min at 4 °C. Flow cytometry was performed using a FACSCalibur (BD Biosciences), collecting 20,000 events. The DNA content (propidium iodide) and DNA synthesis (BrdU content) were analyzed using two parameter data collection using CellQuest (BD Biosciences) software. ModFit LT software (Verity Software House, Topsham, ME, USA) was employed to perform a single parameter analysis of the cell cycle distribution.

### 4.5. Immunohistochemistry

Immunohistochemistry (IHC) was performed with paraffin-embedded tumor tissue following fixation in 10% buffered formalin as detailed elsewhere [13]. The slides were processed as described previously with some modifications (Cell Signaling, Danvers, MA, USA). Antigen unmasking was performed by subjecting the sections to 80 °C for 10 min in 10 mM sodium citrate, pH 6.0. After washing with distilled water, the slides were treated with 3% H_2_O_2_ and 100 µL of PBS containing Tween 20 (PBST) and normal goat serum (5%). Thereafter, γH_2_AX and caspase-3 antibodies (Cell Signaling) in PBST with normal goat serum (5%) were added to the sections and incubated overnight at 4 °C. After washing, the tissue sections were treated with 100 µL DAB (3,3′-diaminobenzidine) substrate, washed, dehydrated in 95% and 100% ethanol, and dipped in xylene. Coverslips were mounted using Cytoseal XYL (Thermo Scientific, Cincinnati, OH, USA).

### 4.6. Immunoblotting

Immunoblot analysis following standard procedures was executed with total protein isolates using T-PER tissue protein extraction reagent (Thermo Scientific) with protease inhibitors (Roche, Indianapolis, IN, USA). Fifty micrograms of total protein per lane was separated via a 4%–20% tris-glycine gel. After transferring to a nitrocellulose membrane, mAbs against cleaved caspase-3, cPARP, γH_2_AX (Cell Signaling), Rad51, and DNA-PKcs (Abcam, Cambridge, MA, USA) containing 5% BSA and 0.05% Tween 20 were used at a dilution of 1:1000 in PBS. Horseradish peroxidase conjugated rabbit secondary antibodies in PBS containing 3% non-fat dry milk were employed at 1:5000. Blots were visualized applying the ECL Plus chemoluminescent detection kit (GE Healthcare) and the images captured by Fuji LAS 4000 (Fujifilm, Stamford, CT, USA).

### 4.7. Metaphase Spread

Tumor tissue was washed with PBS to remove any blood and debris. Supernatant with suspended cells was transferred from the minced tumor tissues. Re-suspended cells in DMEM were incubated for 30 min with colcemid, followed by hypotonic 0.75 M KCl solution for 30 min at 37 °C. Fixation solution (MeOH:glacial acetic acid, 3:1) was slowly added into swollen cells. After centrifugation (100× *g*, 5 min), the cells were re-suspended in fixation solution (10 mL). The cells were again washed with more fixative, collected in 1 mL of fixation solution, spread on microscope slides, and humidified with water steam. Chromosomes were stained with Giemsa solution and mounted with permount.

### 4.8. RNA Purification

Total RNA was isolated from tumor tissues as previously described using the RNeasy mini kit (Qiagen, Hilden, Germany) and held at −80 °C until used for gene expression qRT-PCR arrays as detailed elsewhere [14]. Purity of the isolated total RNA was assayed using a Nano-drop (Thermo Scientific, Wilmington, DE, USA) spectrophotometer and PCR with β-actin primers. Only total RNA possessing an A260/A280 ratio > 1.9 lacking detectable DNA contamination (PCR) was employed in the gene expression array (qRT-PCR array).

### 4.9. Human DNA Damage PCR Array

The cDNA was reversed transcribed from RNA employing the First strand cDNA synthesis Kit (SABiosciences, Frederick, MD, USA). Comparison of relative expression of 84 DNA damage related genes was determined by use of a human DNA damage PCR array (SABiosciences) and the RT^2^ real-time SYBR Green/Rox PCR master mix (SABiosciences) utilizing a 7500 real time PCR system (Applied Biosystems, Rockville, MD, USA). The array encompasses genes associated with apoptosis, cell cycle and damaged DNA binding, and with DNA repair (Table S1).

### 4.10. Statistics

A minimum of three independent experiments were performed for each data point reported in these studies. All values were reported and recorded as the mean ± S.D. Student’s *t*-test was employed for paired data, and multiple statistical comparisons were performed employing ANOVA. A *p*-value <0.05 was considered statistically significant.

## 5. Conclusions

The development of new tools and the examination of the fundamental molecular events that transpire during these combination therapies are expected to aid in making educated choices to take advantage of synergistic mechanisms. Toward these purposes, understanding the cellular and biochemical mechanisms of cell death and DNA repair associated with targeted radiation is important for devising better therapies and reducing adverse effects in normal tissues exposed to irradiation during therapy, or inadvertently because of exposure from environmental sources or nuclear devices. Sequential RIT with ^177^Lu- and ^211^At-labeled BR96 was recently studied in a rat colon carcinoma model [45]. Directly combining α-emitters and β^−^-emitters should be beneficial because such a therapy would take advantage of the different modes of actions of two types radiation (high- and low-LET) as well as then the range of penetration into tumor to effectively complement one another to achieve a higher efficiency in terms of tumor cell eradication. Identifying genes pivotal in tumor responses may also provide information useful for optimization of combination therapies. Optimization of all of the components and variables associated with the development of combination therapies will ultimately improve efficacy and also reduce toxicity. Such goals might possibly be met through the execution of carefully planned pre-clinical investigation combined with improved molecularly targeted strategies to facilitate translation forward into clinical evaluation.

## Figures and Tables

**Table 1:** Cell cycle distribution in LS-174T intraperitoneal (i.p.) tumor xenografts following treatment with ^177^Lu-trastuzumab.

Treatment	Phase	Time Point (h)										
		0	6	24	48	72	96	120	144	168	192	336
None	G1	76.2 ± 1.3										
	S	15.4 ± 1.2										
	G2/M	8.5 ± 0.1										
^177^Lu-trastuzumab	G1		75.7 ± 6.6	76.0 ± 3.9	77.8 ± 3.9	74.9 ± 2.9	78.6 ± 2.1	77.9 ± 5.0	82.5 ± 3.7	77.7 ± 2.6	80.2 ± 3.3	84.4 ± 3.2
	S		15.0 ± 5.7	10.0 ± 3.5	6.10 ± 1.6	4.60 ± 1.1	4.7 ± 0.7	3.6 ± 0.5	3.4 ± 0.5	3.3 ± 0.5	3.5 ± 0.5	2.5 ± 0.1
	G2/M		9.4 ± 3.1	14.0 ± 2.4	16.1 ± 2.6	20.5 ± 2.0	16.6 ± 1.9	18.5 ± 4.8	14.1 ± 3.9	19.1 ± 2.5	16.3 ± 2.9	13.1 ± 3.1
^177^Lu-HuIgG	G1		73.1 ± 5.5	74.7 ± 2.0	77.0 ± 5.0	71.8 ± 4.4	75.5 ± 1.7	78.0 ± 3.5	80.2 ± 4.9	76.2 ± 4.6	77.6 ± 3.1	75.0 ± 2.7
	S		18.8 ± 3.2	10.6 ± 3.1	6.60 ± 2.0	8.30 ± 4.4	7.9 ± 3.6	6.4 ± 1.2	7.3 ± 2.5	11.1 ± 2.8	9.5 ± 2.9	12.9 ± 3.0
	G2/M		8.1 ± 3.3	14.7 ± 2.0	16.3 ± 6.0	19.9 ± 8.5	16.6 ± 4.2	15.7 ± 3.0	12.5 ± 2.7	12.7 ± 2.5	12.9 ± 1.2	12.2 ± 3.4

Results represent the average of a minimum of three replications ± standard deviation (SD). Values represent a percentage.

**Table 2:** Expression of genes involved in apoptosis in LS-174T i.p. xenografts following treatment with ^177^Lu-trastuzumab.

Symbol	GeneBank ID	Fold Change							
		24 h				168 h			
		^177^Lu-Trastuzumab	*p*	^177^Lu-HuIgG	*p*	^177^Lu-Trastuzumab	*p*	^177^Lu-HuIgG	*p*
*ABL*	NM_005157	−1.6	0.001	−2.2	0.036	4.9	0.012	−2.6	0.001
*BRCA1*	NM_007294	−2.9	0.013	−3.2	0.011	−6.6	0.010	−4.8	0.006
*CIDEA*	NM_001279	−7.0	0.001	−3.3	0.002	1.8	0.219	−4.3	0.001
*GADD45α*	NM_001924	1.8	0.007	1.9	0.040	2.2	0.013	3.7	0.000
*GADD45γ*	NM_006705	1.2	0.669	1.3	0.690	2.5	0.050	2.1	0.040
*GML*	NM_002066	−1.6	0.002	−2.2	0.046	5.0	0.010	−2.7	0.008
*IP6K3*	NM_054111	−1.1	0.931	−1.2	0.899	5.0	0.010	−1.6	0.210
*PCBP4*	NM_020418	1.1	0.439	1.6	0.061	2.1	0.110	3.3	0.000
*RAD21*	NM_006265	−1.2	0.212	−1.3	0.198	−2.8	0.011	−2.4	0.010
*p73*	NM_005427	−1.0	0.962	−1.7	0.936	2.8	0.023	2.6	0.026

Mice bearing i.p. LS-174T xenografts were treated with ^177^Lu-trastuzumab for 24 and 168 h. qRT-PCR array was used for gene expression analysis in three independent experiments. The number indicates the fold change (>2-fold) compared to the untreated control. Additional groups include a non-specifically targeted control, ^177^Lu-HuIgG. Results represent the average of a minimum of three replicates.

**Table 3:** Expression of genes involved in cell cycle in LS-174T i.p. xenografts following treatment with ^177^Lu-trastuzumab.

Symbol	GeneBank ID	Fold Change							
		24 h				168 h			
		^177^Lu-Trastuzumab	*p*	^177^Lu-HuIgG	*p*	^177^Lu-Trastuzumab	*p*	^177^Lu-HuIgG	*p*
*BRCA1*	NM_007294	−2.9	0.013	−3.2	0.011	−6.6	0.010	−4.8	0.006
*CHK1*	NM_001274	−1.7	0.004	−1.9	0.001	−4.6	0.001	−3.7	0.001
*CHK2*	NM_007194	−1.7	0.023	−1.9	0.010	−7.1	0.001	−2.5	0.003
*DDIT3*	NM_004083	−2.5	0.008	−2.9	0.006	−2.5	0.053	−1.6	0.037
*FANCG*	NM_004629	−2.6	0.003	−2.4	0.004	−1.7	0.022	−4.3	0.001
*GADD45α*	NM_001924	1.8	0.007	1.9	0.040	2.2	0.013	3.7	0.000
*GML*	NM_002066	−1.6	0.001	−2.2	0.046	5.0	0.010	−2.7	0.008
*GTSE1*	NM_016426	−1.7	0.032	−2.4	0.009	−9.0	0.001	−3.5	0.004
*MAPK12*	NM_002969	−4.7	0.005	−5.0	0.005	1.5	0.053	−3.6	0.007
*NBN*	NM_002485	−1.1	0.037	−1.2	0.035	−2.8	0.015	−1.3	0.731
*PCBP4*	NM_020418	1.1	0.043	1.5	0.061	2.1	0.116	3.2	0.000
*SESN1*	NM_014454	4.5	0.001	5.4	0.000	2.3	0.008	4.0	0.002

Mice bearing i.p. LS-174T xenografts were treated with ^177^Lu-trastuzumab for 24 and 168 h. qRT-PCR array was used for gene expression analysis in three independent experiments. The number indicates the fold change (>2-fold) compared to the untreated control. Additional groups include a non-specifically targeted control, ^177^Lu-HuIgG. Results represent the average of a minimum of three replicates.

**Table 4:** Expression of genes involved in DNA damage repair in LS-174T i.p. xenografts following treatment with ^177^Lu-trastuzumab.

Symbol	GeneBank ID	Fold Change							
		24 h				168 h			
		^177^Lu-Trastuzumab	*p*	^177^Lu-HuIgG	*p*	^177^Lu-Trastuzumab	*p*	^177^Lu-HuIgG	*p*
*BRCA1*	NM_007294	−2.9	0.013	−3.2	0.010	−6.6	0.010	−4.8	0.006
*BTG2*	NM_006763	3.4	0.000	4.8	0.000	3.9	0.004	4.8	0.000
*ERCC1*	NM_001983	1.1	0.486	1.2	0.491	1.8	0.160	2.8	0.000
*EXO1*	NM_130398	−1.8	0.061	−2.4	0.014	−3.4	0.008	−3.8	0.006
*FANCG*	NM_004629	−2.6	0.002	−2.4	0.004	−1.7	0.022	−4.3	0.001
*FEN1*	NM_004111	−1.4	0.219	−2.1	0.085	−4.7	0.023	−2.2	0.059
*LIG1*	NM_00234	−1.9	0.009	−1.6	0.015	−1.5	0.048	−3.3	0.012
*MRE11A*	NM_005590	2.4	0.001	1.2	0.434	−3.7	0.006	−3.6	0.035
*MSH2*	NM_00251	−1.2	0.229	−1.6	0.081	−5.4	0.014	−2.1	0.030
*NBN*	NM_002485	−1.1	0.392	−1.2	0.357	−2.8	0.015	−1.3	0.731
*PNKP*	NM_007254	−3.2	0.006	−2.7	0.011	1.9	0.003	−1.8	0.027
*PRKDC*	NM_006904	−1.1	0.392	−1.0	0.933	−5.4	0.002	−1.7	0.016
*RAD18*	NM_020165	−2.1	0.014	−2.5	0.006	−1.5	0.006	−3.3	0.002
*RAD21*	NM_006265	−1.1	0.007	−1.3	0.198	−2.8	0.011	−2.4	0.010
*RAD51B*	NM_133509	−1.6	0.372	−1.8	0.897	−5.0	0.018	−1.8	0.040
*p73*	NM_005427	−1.0	0.962	−1.7	0.936	2.8	0.023	2.6	0.026
*UNG*	NM_003362	−2.3	0.002	−1.9	0.014	−1.7	0.024	−2.4	0.001
*XPC*	NM_004628	2.7	0.001	3.5	0.003	2.3	0.003	3.0	0.000
*XRCC2*	NM_005431	1.0	0.988	−1.3	0.270	−1.3	0.271	−2.6	0.031

Mice bearing i.p. LS-174T xenografts were treated with ^177^Lu-trastuzumab for 24 and 168 h. qRT-PCR array was used for gene expression analysis in three independent experiments. The number indicates the fold change (>2-fold) compared to the untreated control. Additional groups include a non-specifically targeted control, ^177^Lu-HuIgG. Results represent the average of a minimum of three replicates.

## Supplementary Materials

Supplementary materials can be found at http://www.mdpi.com/1422-0067/17/5/736/s1.
