# Supplementary material for: Mechanisms of Cell Killing Response from Low Linear Energy Transfer (LET) Radiation Originating from 177Lu Radioimmunotherapy Targeting Disseminated Intraperitoneal Tumor Xenografts
Source: Int J Mol Sci. 2016 May 16;17(5):736. doi: 10.3390/ijms17050736 (PMC4881558; doi:10.3390/ijms17050736)
Supplement: Supplementary file 1 [file ijms-17-00736-s001.ppt]

## Slide 1
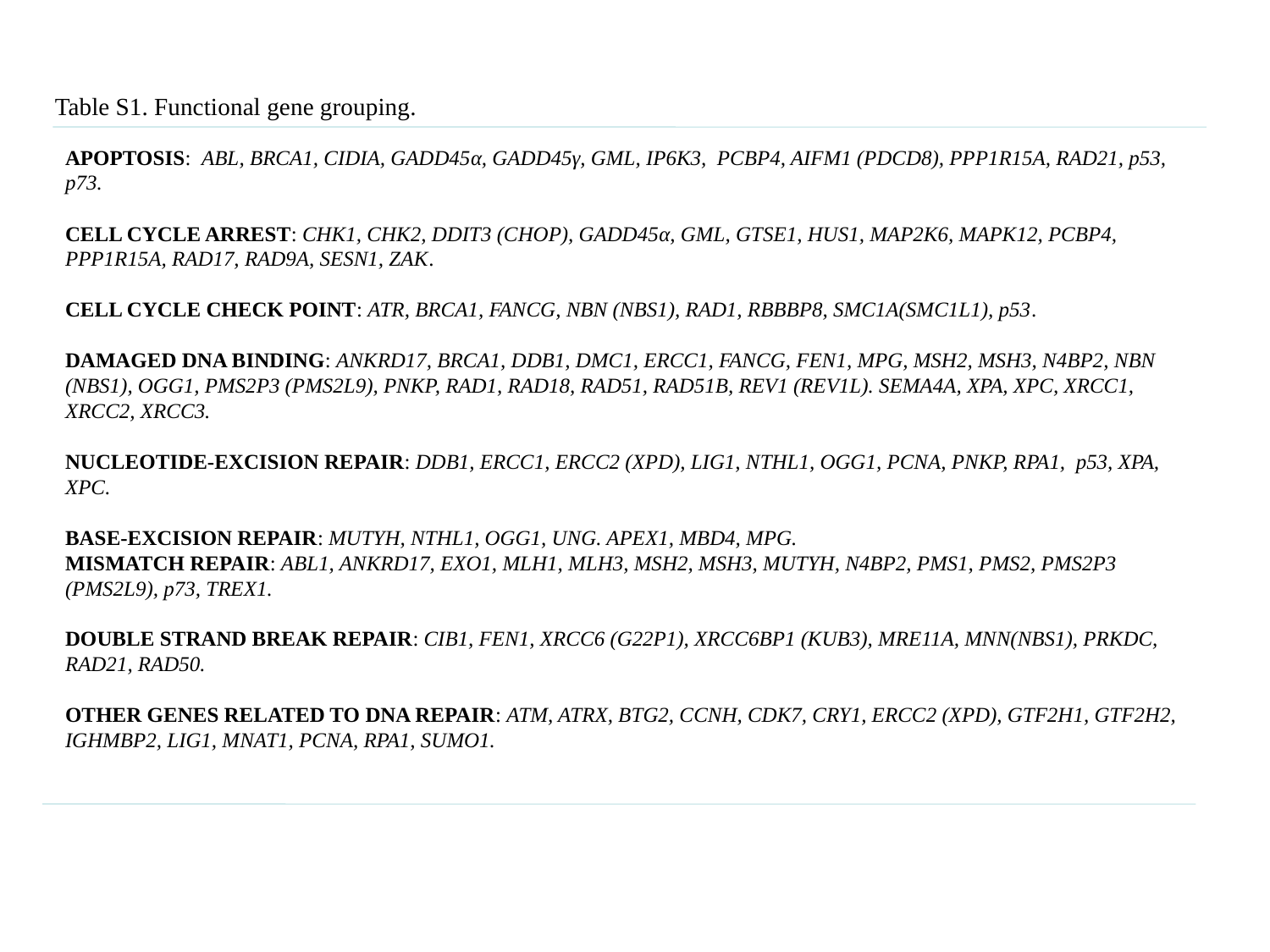

Table S1. Functional gene grouping.
APOPTOSIS: ABL, BRCA1, CIDIA, GADD45α, GADD45γ, GML, IP6K3, PCBP4, AIFM1 (PDCD8), PPP1R15A, RAD21, p53, p73.
CELL CYCLE ARREST: CHK1, CHK2, DDIT3 (CHOP), GADD45α, GML, GTSE1, HUS1, MAP2K6, MAPK12, PCBP4, PPP1R15A, RAD17, RAD9A, SESN1, ZAK.
CELL CYCLE CHECK POINT: ATR, BRCA1, FANCG, NBN (NBS1), RAD1, RBBBP8, SMC1A(SMC1L1), p53.
DAMAGED DNA BINDING: ANKRD17, BRCA1, DDB1, DMC1, ERCC1, FANCG, FEN1, MPG, MSH2, MSH3, N4BP2, NBN (NBS1), OGG1, PMS2P3 (PMS2L9), PNKP, RAD1, RAD18, RAD51, RAD51B, REV1 (REV1L). SEMA4A, XPA, XPC, XRCC1, XRCC2, XRCC3.
NUCLEOTIDE-EXCISION REPAIR: DDB1, ERCC1, ERCC2 (XPD), LIG1, NTHL1, OGG1, PCNA, PNKP, RPA1, p53, XPA, XPC.
BASE-EXCISION REPAIR: MUTYH, NTHL1, OGG1, UNG. APEX1, MBD4, MPG.
MISMATCH REPAIR: ABL1, ANKRD17, EXO1, MLH1, MLH3, MSH2, MSH3, MUTYH, N4BP2, PMS1, PMS2, PMS2P3 (PMS2L9), p73, TREX1.
DOUBLE STRAND BREAK REPAIR: CIB1, FEN1, XRCC6 (G22P1), XRCC6BP1 (KUB3), MRE11A, MNN(NBS1), PRKDC, RAD21, RAD50.
OTHER GENES RELATED TO DNA REPAIR: ATM, ATRX, BTG2, CCNH, CDK7, CRY1, ERCC2 (XPD), GTF2H1, GTF2H2, IGHMBP2, LIG1, MNAT1, PCNA, RPA1, SUMO1.
